# Psychosocial impact of cervical cancer diagnosis: a study conducted in the Radiotherapy Department at CHU Hassan II, Fez, Morocco

**DOI:** 10.3332/ecancer.2025.1976

**Published:** 2025-08-29

**Authors:** Khalfi Samia, Benhaddouch Yassine, Sadiki El Mehdi, Hassani Wissal, Farhane Fatima Zahra, Alami Zenab, Bouhafa Touria

**Affiliations:** 1 Faculty of Medicine and Pharmacy of Fez, Hassan II University Hospital, Sidi Mohammed Ben Abdellah University,Fez, Morocco, 30000; 2 Laboratory of Applied Physics, Computer Science and Statistics, Faculty of Sciences Dhar El Mahraz, Sidi Mohammed Ben Abdellah University, Fez, Morocco 30000; 3 Research Team for Mental Health, University Psychiatric Hospital, Tangier, Morocco 90000; 4 Faculty of Medicine and Pharmacy of Tangier, Abdelmalek Essaadi University, Tangier, Morocco 90000

**Keywords:** cervical cancer, diagnosis, psychosocial impact

## Abstract

**Background:**

Cervical cancer is a major public health issue in Morocco, ranking as the second most common cancer among women. Beyond its physical burden, a diagnosis of cervical cancer significantly affects patients emotionally and socially, often leading to anxiety, depression and social isolation. Despite the high prevalence of this cancer, limited research has explored its psychosocial impact within the Moroccan context.

**Methods:**

This study assessed the psychosocial impact of cervical cancer diagnosis among women treated at the Radiotherapy Department of Hassan II University Hospital in Fez. Emotional distress was evaluated using the Hospital Anxiety and Depression Scale (HADS), while quality of life (QoL) was measured using the WHOQOL-BREF tool. Coping strategies were analysed using the Brief Cope Inventory. All tools were validated in Moroccan Arabic dialect for cultural relevance.

**Results:**

A total of 100 patients were enrolled. Emotional distress was notable, with 38% of participants experiencing significant anxiety (HADS score >8) and 25% reporting symptoms of depression. QoL scores (WHOQOL-BREF) were moderate, with significant deficits in physical and psychological health domains. Coping strategies predominantly included religious practices and family support, which correlated with better management of emotional distress. Social consequences, such as marital tension and isolation, were frequently reported but were mitigated by robust familial support.

**Conclusion:**

The study highlights the profound psychosocial burden of cervical cancer diagnosis in Moroccan women. Integrated support programs encompassing physical, emotional, social and spiritual care are urgently needed to improve patient outcomes. Future research should focus on culturally tailored interventions to enhance coping mechanisms and overall QoL.

## Introduction

Cervical cancer remains a major public health concern worldwide, particularly in low- and middle-income countries. In Morocco, it is the second most frequent cancer among women, with approximately 1,500 new cases diagnosed annually [1, 2]. Beyond its physical impact, cervical cancer poses significant psychological and social challenges, particularly in culturally sensitive contexts [3]. A diagnosis often leads to anxiety, depression, social isolation and strained familial relationships, underscoring the need for comprehensive care that addresses both physical and psychosocial dimensions [4, 5].

While numerous studies have explored the psychosocial burden of cervical cancer globally, there is limited evidence from North African settings, including Morocco [6]. This study aims to fill this gap by evaluating the emotional, social and quality-of-life (QoL) impacts of cervical cancer diagnosis in a Moroccan cohort. Additionally, it investigates coping mechanisms employed by patients, providing insights for culturally relevant interventions.

## Methods

### Study design and setting

This study employed a cross-sectional design and was conducted at the Radiotherapy Department of CHU Hassan II, Fez, Morocco. The department serves a diverse population from both urban and rural regions, providing an ideal setting to assess the psychosocial impact of cervical cancer in a Moroccan context. The study focused on women undergoing treatment for newly diagnosed cervical cancer between January and June 2024.

### Participants

Inclusion criteria included adult patients (aged 18 to 75 years) diagnosed with cervical cancer, with at least 6 months of post-treatment follow-up, not undergoing active cancer treatment (chemotherapy, radiotherapy or surgical intervention) at the time of the study, and having signed an informed consent. Patients were excluded if they had recurrent disease, cognitive impairments or comorbidities that could confound the assessment of psychosocial outcomes. The comorbidities excluded were severe psychiatric disorders (e.g., schizophrenia and bipolar disorder), significant neurological conditions and uncontrolled chronic illnesses (such as severe cardiovascular or respiratory diseases) that could interfere with the assessment of anxiety, depression and QoL. Participants were recruited through consecutive sampling, and the sample size was determined based on the prevalence of cervical cancer in Morocco and the available resources for the study. This sample allowed for the collection of meaningful data while remaining feasible within the local context.

### Data collection

Data collection involved structured interviews conducted by trained healthcare professionals fluent in Moroccan Arabic. Three validated tools were used to gather comprehensive data:

Hospital Anxiety and Depression Scale (HADS): This 14-item questionnaire was employed to assess emotional distress, specifically anxiety and depression. Scores above eight indicated clinically significant symptoms. The tool’s adaptation to Moroccan Arabic ensured cultural relevance and comprehensibility.

WHOQOL-BREF: This 26-item instrument measured QoL across four domains: physical health, psychological health, social relationships and environmental factors. Higher scores indicated better QoL. The tool was administered in its culturally adapted version.

Brief Cope Inventory: This 28-item questionnaire evaluated coping strategies categorised into problem-focused, emotion-focused and avoidance-oriented approaches. Religious coping, a culturally significant strategy, was specifically emphasised in this cohort.

### Ethical considerations

Ethical approval was obtained. Written informed consent was secured from all participants. Confidentiality and anonymity were maintained throughout the study.

### Data analysis

Quantitative data were entered into a secure database and analysed using R statistical software. Descriptive statistics summarised demographic and clinical characteristics, while inferential statistics explored associations between emotional distress, QoL and coping strategies. Univariate analysis was conducted using chi-square tests for qualitative variables and Student’s *t*-tests or ANOVA for quantitative variables, to assess associations between participant characteristics and anxiety, depression or QoL scores. For multivariate analysis, multiple linear regression models were used. The dependent variables were anxiety, depression (HADS) and QoL scores. The independent variables included education level, employment status, marital status, monthly income, cancer stage and type of treatment.

## Results

### Demographic and clinical characteristics

The study enrolled 100 patients with a mean age of 47 years (± 9). The majority of participants were married (60%) and with a low income. Table 1 summarises the demographic and clinical characteristics of the cohort.

### Social consequences

Commonly reported issues included marital tensions (15%) and social isolation (10%). Additionally, 20% of participants mentioned experiencing significant marital problems, while 60% reported receiving emotional support from their families.

### Emotional impact

Emotional distress was prevalent among the participants, with 38% exhibiting significant anxiety (HADS > 8) and 25% reporting symptoms of depression.

In univariate analysis, higher levels of anxiety and depression were observed among married women, housewives, those with low income and patients with locally advanced stages of the disease (Table 2).

In multivariate analysis, only low income and advanced disease stage significantly impacted anxiety and depression levels (Table 3).

Women with limited family support were more likely to experience higher levels of emotional distress. Patients with significant fear of recurrence had higher mean scores for anxiety (8.2) and depression (7.4), with statistically significant results (*p* = 0.03 for anxiety, *p* = 0.04 for depression). Conversely, social support did not have a significant impact on these scores (*p* = 0.15 for anxiety, *p* = 0.20 for depression), suggesting that fear of recurrence is a major predictive factor in the intensification of anxiety and depressive symptoms among these patients (Table 4).

### Quality of life

The QoL, as measured by WHOQOL-BREF, showed moderate scores across all domains, with physical and psychological health being the most affected. The results reveal a significant correlation between HADS scores and the domains affected by these emotional disorders Table 5.

### Coping strategies

**Figure 1.**
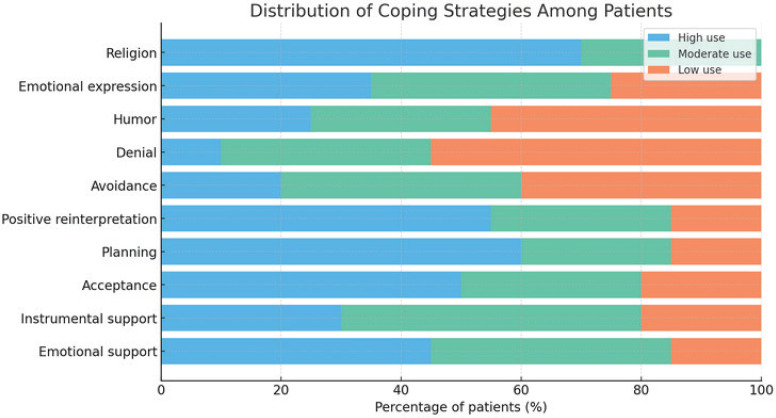
Distribution of coping strategies among patients.

The majority of patients employ active coping strategies, including seeking social support and planning. However, a particularly striking aspect is the prominence of religious coping strategies: 85% of patients turn to spiritual practices, such as prayer, to manage anxiety and depression associated with the fear of recurrence, followed by reliance on family support. These approaches were linked to lower levels of anxiety and depression Figure 1.

## Discussion

The results of this study provide valuable insights into the psychosocial impact of cervical cancer diagnosis among women in Morocco. Emotional distress, including anxiety and depression, is prevalent in this cohort, with a significant proportion of patients reporting clinically significant symptoms. The moderate QoL scores across physical, psychological and social domains highlight the substantial burden of the disease on patients' overall well-being. The study's findings also underline the important role of coping strategies, particularly spirituality and familial support, in mitigating emotional distress and promoting better psychosocial outcomes.

Cervical cancer significantly impacts the mental health of patients, with anxiety and depression being prevalent psychological issues. Studies indicate that these psychiatric morbidities are common among women undergoing treatment for cervical cancer, influenced by various factors such as disease stage, treatment type and personal circumstances. The prevalence of anxiety and depression underscores the need for comprehensive psychosocial care for these patients.

A study involving 30 women treated for cervical cancer found that 56.6% exhibited pathological levels of anxiety and depression, with factors such as marital discord and decreased sexual activity post-treatment contributing significantly to these conditions [7].

Another study of 40 Tunisian women in remission reported that 60% experienced anxiety and 57.5% experienced depression, with higher rates observed in those with advanced disease stages and those who underwent hysterectomy or external radiotherapy [8].

In a larger cohort of 83 patients, 75.7% of fertile-aged women experienced clinically significant anxiety, while 57.5% had clinically expressed depression [9].

A Korean study aimed to assess the prevalence of anxiety and depression among 828 cervical cancer survivors and to identify associated factors. The study found an anxiety prevalence of 39.5%, particularly high among women aged 50 or younger, and a depression prevalence of 34.6%, significantly lower than that of the control group. The main factors associated with these psychological symptoms included financial difficulties, poor body image, sexual inactivity and low existential well-being. Anxiety was also linked to insomnia and lack of social support, while depression was associated with older age and impaired social functioning. No significant association was found with clinical characteristics of the cancer [10].

A French population-based study assessed cancer-related fatigue, anxiety and QoL in long-term survivors of breast, cervical and colorectal cancer, approximately 15 years post-diagnosis. Compared to cancer-free controls, survivors reported significantly higher levels of fatigue—particularly general and mental fatigue—as well as elevated anxiety levels. Interestingly, despite these persistent symptoms, their overall QoL was comparable to that of the general population. These findings highlight the long-term psychological and physical burden experienced by cancer survivors, underscoring the need for ongoing supportive care well beyond the initial treatment phase [11]. The QoL for patients diagnosed with locally advanced cervical cancer is significantly impacted by the disease and its treatment. Research indicates that while some patients experience improvements post-treatment, many continue to face long-term challenges affecting various aspects of their lives.

Advanced cervical cancer leads to a substantial decline in health-related QoL, with studies reporting average utility values around 0.4653. Common issues include pain (42.2%) and emotional distress (29.6%) [12]. A study showed that patients often present with poor QoL at diagnosis, with 60.5% classified as having a poor QoL [13].

The moderate QoL scores observed in our study, particularly in the physical and psychological domains, highlight the significant impact of cervical cancer on various aspects of patients' lives. Additionally, our study reveals a correlation between emotional distress (measured by HADS scores) and lower scores in physical and psychological health domains, reinforcing the well-established link between mental health and QoL in cancer patients.

The psychological impact of a cervical cancer diagnosis, especially in developing countries, is profound and complex, often involving significant emotional distress, stigma and social discrimination that worsen mental health challenges. Initial reactions to the diagnosis typically include shock, fear and confusion, leading to anxiety and depression [14]. Women often experience feelings of shame and self-blame, particularly due to societal stigma related to sexually transmitted infections like HPV [14]. Social discrimination is widespread, with 61.8% of women reporting social exclusion and 89% feeling a loss of femininity. Additionally, economic hardships are common, with 71% of women facing financial strain due to medical costs, which affects their overall QoL [15].

To cope, many women make lifestyle changes, adopt healthier behaviours and seek spiritual support. Psychotherapy and support groups are vital in addressing the psychological needs of survivors. While the psychological impact is significant, some women find empowerment through their diagnosis, leading to greater awareness and proactive health management. This duality underscores the complexity of coping with cervical cancer in these contexts [15, 16].

However, what distinguishes this study is the cultural context of Morocco, where religious and familial coping strategies play a particularly central role. The prominence of religious coping strategies in this cohort (85% of participants reported using prayer) is noteworthy and reflects the strong influence of spirituality in Moroccan society.

The comparative study of religious coping methods among cancer patients in Malaysia, Iran and Turkey highlights the significant cultural impact of Islamic beliefs on coping strategies. While these countries share a common religious framework, the application of religious coping methods differs, revealing the interplay between culture and religion in cancer care.

In Malaysia, patients often rely on communal prayers and seek support from family and religious leaders, emphasising collective coping strategies. In Iran, the focus shifts toward personal faith and individual prayer, with a strong emphasis on spiritual counseling and dhikr (remembrance of God) as coping mechanisms [17, 18]. In Turkey, patients experience a mix of spiritual distress and well-being, using religious practices to foster acceptance and resilience during treatment [19].

Spiritual beliefs can play an important role in accepting a cancer diagnosis, providing hope and emotional support, particularly in couples, where shared faith can strengthen coping mechanisms [20]. Some studies highlight the importance of spiritual care interventions, such as religious-spiritual psychotherapy and support groups based on spiritual teachings, in offering holistic care to cancer patients [18, 21]. However, the specific impact of these practices has not been systematically assessed in this study.

Furthermore, the study's findings regarding social consequences such as marital tensions and isolation echo those reported by other researchers, such as Nalbant *et al* [22], who found that cancer diagnosis often leads to disrupted relationships. However, in this study, the impact of these social consequences was mitigated by familial support, with 60% of participants reporting emotional support from their families. This finding reinforces the notion that family remains a critical source of coping and emotional resilience in Moroccan women with cervical cancer [22].

### Strengths of the study

This study's strengths lie in its rigorous methodology, which included validated tools for assessing emotional distress (HADS), QoL (WHOQOL-BREF) and coping strategies (Brief Cope Inventory) in a culturally adapted format. The use of structured interviews with trained professionals ensures that data were collected in a reliable and consistent manner. The inclusion of both urban and rural patients, as well as the focus on newly diagnosed patients undergoing radiotherapy, enhances the generalisability of the findings within the Moroccan context.

Additionally, the study highlights the importance of considering cultural and social factors when assessing psychosocial outcomes in cancer patients. By focusing on the specific coping strategies employed by Moroccan women, such as religious practices and family support, this research provides valuable information for the development of culturally relevant interventions that can better address the needs of these patients.

### Limitations of the study

One limitation of this study is its cross-sectional design, which prevents the assessment of causal relationships between variables. Longitudinal studies would be beneficial to track the psychosocial trajectory of cervical cancer patients over time, particularly in relation to their coping mechanisms and QoL during and after treatment.

Another limitation is the relatively small sample size (100 patients), which may affect the statistical power of some analyses. Larger studies would be needed to confirm the findings and explore potential subgroup differences based on sociodemographic characteristics, such as education level and income.

## Conclusion and perspectives

This study underscores the significant psychosocial burden of cervical cancer on Moroccan women, highlighting the emotional distress experienced by patients and the critical role of coping strategies in managing anxiety and depression. The findings emphasise the need for integrated care models that address not only the physical aspects of the disease but also the emotional, social and spiritual needs of patients.

Future research should focus on exploring the effectiveness of culturally tailored interventions aimed at improving coping strategies, such as religious or family-based support systems. Interventions that incorporate psychological counseling, social support networks and spiritual practices could offer holistic support to cervical cancer patients in Morocco and similar cultural contexts. Furthermore, longitudinal studies that track patients' psychosocial outcomes over time will provide more insights into how coping strategies evolve and how they influence long-term QoL.

## Conflicts of interest

The authors declare that they have no conflicts of interest related to this study.

## Funding

The authors received no financial support or external funding for the research.

## Figures and Tables

**Table 1.** Demographic and clinical characteristics.

Variable	Effect (*n*)	Percentage (%)
Mean age	47 years (± 9)	10%
Education level	Illiterate	45%
	Primary or less	30%
	Secondary	15%
	University	10%
Marital status	Married	60%
	Single	10%
	Widowed	5%
	Divorced	25%
Monthly income	Low	60%
	Medium	30%
	High	10%
Geographic origin	Urban	65%
	Rural	35%
Professional status	Homemaker	55%
	Unskilled worker	25%
	Skilled worker	10%
	Unemployed	10%
Stage of disease	Stage I–II	25%
	Stage III	40%
	Stage IV	35%
Treatment type	Radiochemotherapy + Brachytherapy	80%
	Surgery + External beam radiotherapy	10%
	Surgery + Brachytherapy	8%
	Radiotherapy alone	2%

**Table 2.** Univariate analysis of sociodemographic and clinical factors associated with anxiety and depression.

Demographic variable	Category	Anxiety (HADS) – Mean ± SD	Depression (HADS) – Mean ± SD	*p*-value anxiety (*)	*p*-value depression (*)
Education level	Illiterate	12.0 ± 3.5	10.5 ± 2.9	*p* = 0.12	*p* = 0.09
	Primary or less	11.0 ± 3.0	10.2 ± 2.7	*p* = 0.05	*p* = 0.07
	Secondary	10.8 ± 3.1	9.9 ± 2.6	*p* = 0.08	*p* = 0.10
	University	9.8 ± 2.7	9.4 ± 2.5	*p* = 0.13	*p* = 0.15
Marital status	Married	10.2 ± 3.1	9.7 ± 2.8	*p* = 0.03	*p* = 0.04
	Single	12.5 ± 3.6	11.0 ± 3.2	*p* = 0.04	*p* = 0.05
	Widowed	11.8 ± 3.3	10.7 ± 2.9	*p* = 0.11	*p* = 0.12
	Divorced	11.0 ± 3.0	10.0 ± 2.7	*p* = 0.06	*p* = 0.08
Monthly income	Low (< 3,000 MAD)	12.0 ± 3.5	11.2 ± 3.0	*p* = 0.02	*p* = 0.03
	Medium (3,000–6,000 MAD)	10.5 ± 3.0	9.8 ± 2.7	*p* = 0.05	*p* = 0.06
	High (> 6,000 MAD)	9.2 ± 2.8	9.1 ± 2.4	*p* = 0.09	*p* = 0.12
Geographic origin	Urban	10.5 ± 3.1	10.0 ± 2.8	*p* = 0.04	*p* = 0.06
	Rural	12.0 ± 3.6	11.0 ± 3.2	*p* = 0.05	*p* = 0.07
Employment status	Housewife	12.5 ± 3.7	11.5 ± 3.0	*p* = 0.01	*p* = 0.02
	Unskilled worker	11.5 ± 3.3	10.2 ± 2.8	*p* = 0.04	*p* = 0.06
	Skilled worker	10.5 ± 3.1	9.7 ± 2.6	*p* = 0.07	*p* = 0.10
	Unemployed	13.0 ± 3.9	12.0 ± 3.5	*p* = 0.02	*p* = 0.03
Disease stage	Stage I–II	9.5 ± 2.8	9.2 ± 2.5	*p* = 0.10	*p* = 0.05
	Stage III	11.5 ± 3.2	10.4 ± 2.9	*p* = 0.03	*p* = 0.04
	Stage IV	12.2 ± 3.5	11.0 ± 3.1	*p* = 0.02	*p* = 0.03
Treatment type	Radiochemotherapy + Brachytherapy	11.7 ± 3.3	10.5 ± 2.9	*p* = 0.02	*p* = 0.03
	Surgery + External Radiotherapy	10.5 ± 2.9	9.8 ± 2.6	*p* = 0.07	*p* = 0.08
	Surgery + Brachytherapy	11.0 ± 3.0	10.0 ± 2.7	*p* = 0.06	*p* = 0.09
	Radiotherapy only	13.0 ± 3.8	12.2 ± 3.3	*p* = 0.01	*p* = 0.02

* Bonferroni correction was applied to adjust for multiple comparisons. The adjusted threshold for statistical significance was set at *p* < 0.0036

**Table 3.** Results of multivariate linear regression analyses for anxiety and depression scores (HADS).

Independent variable	Category (reference)	Anxiety (β)	95% CI	*p*-value	Depression (β)	95% CI	*p*-value
Monthly income	Low versus High	**+2.6**	[1.1 ; 4.1]	**0.001**	**+2.3**	[1.0 ; 3.6]	**0.002**
	Medium versus High	+1.1	[–0.2 ; 2.4]	0.09	+0.9	[–0.3 ; 2.1]	0.12
Disease stage	Advanced (III–IV) versus Early (I–II)	**+2.4**	[0.9 ; 3.9]	**0.003**	**+2.0**	[0.8 ; 3.2]	**0.004**
Education level	Illiterate versus University	+1.3	[–0.4 ; 3.0]	0.13	+1.0	[–0.5 ; 2.5]	0.18
Marital status	Single versus Married	+1.0	[–0.2 ; 2.2]	0.10	+0.8	[–0.3 ; 1.9]	0.15
Geographic origin	Rural versus Urban	+0.9	[–0.2 ; 2.0]	0.11	+0.7	[–0.4 ; 1.8]	0.18
Employment status	Housewife versus Employed	+1.2	[–0.1 ; 2.5]	0.07	+1.0	[–0.2 ; 2.2]	0.09
Treatment type	Radiotherapy only versus Combined treatments	+1.1	[–0.3 ; 2.5]	0.10	+1.0	[–0.4 ; 2.4]	0.12

*Bold values indicate statistically significant *p*-values ( *p* < 0.05)

**Table 4.** Prevalence and mean scores of anxiety, depression, fear of recurrence/progression and the impact of social support on these psychological factors in the study population.

Variable	Percentage (%)	Mean HADS anxiety score	Mean HADS depression score	(*p*-value)
Anxiety	38%	11.5 ± 3.2	-	(*p* = 0.03)
Depression	25%	-	10.3 ± 2.8	(*p* = 0.04)
Fear of recurrence/progression	65%	12.0 ± 3.0	-	(*p* = 0.04)
High social support	60%	9.5 ± 2.1	8.5 ± 2.3	(*p* = 0.07)
Low social support	40%	13.2 ± 3.5	12.5 ± 3.1	(*p* = 0.01)

**Table 5.** The mean scores for each domain and their correlation with anxiety and depression.

(WHOQOL-BREF)	Mean Score (out of 100)	Percentage (%)	Correlation with high HADS (Anxiety)	*p*-value (Anxiety)	Correlation with high HADS (Depression)	*p*-value (Depression)
Physical	45 ± 12	Low (70%) Moderate (20%) High (10%)	*r* = −0.43	*p* = 0.02	*r* = −0.50	*p* = 0.01
Psychological	50 ± 10	Low (60%) Moderate (30%) High (10%)	*r* = −0.30	*p* = 0.05	*r* = −0.40	*p* = 0.03
Social	55 ± 9	Low (50%) Moderate (40%) High (10%)	*r* = −0.25	*p* = 0.08	*r* = −0.35	*p* = 0.04
Environmental	54 ± 10	Low (50%) Moderate (40%) High (10%)	*r* = −0.20	*p* = 0.08	*r* = −0.30	*p* = 0.12
Overall satisfaction	60 ± 11	Low (40%) Moderate (40%) High (20%)	*r* = −0.20	*p* = 0.12	*r* = −0.25	*p* = 0.10
